# Supplementary material for: Predicting Frailty and Geriatric Interventions in Older Cancer Patients: Performance of Two Screening Tools for Seven Frailty Definitions—ELCAPA Cohort
Source: Cancers (Basel). 2022 Jan 4;14(1):244. doi: 10.3390/cancers14010244 (PMC8750824; doi:10.3390/cancers14010244)
Supplement: Supplementary file 1 [file cancers-14-00244-s001.zip › cancers-1498399-supplementary.pdf]

Supplementary Table S1. Frailty classification approaches (fit vs. "unfit") and indicators according to the Balducci and International Society of Geriatric Oncology (SIOG) task force classifications.

**Classifications**	**Indicators used to classify patients as fit or unfit**	**Fit**	**Unfit** [a]
***Balducci** & Extermann,2000; Balducci & Beghe, 2000*			
	ADL (Katz)	6/6 and	≤5/6 and/or
	IADL (Lawton)	8/8 and	≤7/8 and/or
	Comorbidities grade 3/4	0 and	≥1 and/or
	Geriatric syndromes [b]	0	≥ 1
***Droz** et al. (SIOG), 2010*			
	ADL (Katz)	6/6 and	≤ 5/6 and/or
	IADL (Lawton) [c]	8/8 and	≤ 7/8 and/or
	Comorbidities grade 3/4	0 and	≥1 and/or
	Malnutrition [d]	absence	at risk/severe

[a] vulnerable, frail or too sick

[b] among dementia (Mini-Mental State Examination score [MMSE]≤23/30), delirium, depression (diagnosed by a semi-structured interview to identify criteria for a major depressive episode from the Diagnostic and Statistical Manual of Mental Disorders [DSM-IV]), urinary and/or fecal incontinence and falls (≥ 1 fall in the last 6 months).

[c] in sensitivity analysis, we considered the 4-item IADL for men: ability to manage money, manage medications, use transportation, and use the telephone.

[d] absence: <10% of weight loss in the past 6 months and <5% in the last month; at risk: 10–15% weight loss in the past 6 months and/or 5–10% in the last month; severe malnutrition: ≥15% weight loss in the past 6 months and/or ≥10% in the last month.

Supplementary Table S2. Variables and definitions used to classify patients in the latent class typology

Variable	Definition
Inadequate social environment	Absence of a primary caregiver or of adequate support at home or of a strong circle of family and friends able to meet the needs of the patient at the time of the evaluation
Malnutrition	One or more of the following criteria as recommended by the French National Authority for Health: at least 10% weight loss in 6 months or 5% in 1 month and/or body mass index < 21 kg/m² and/or Mini-Nutritional Assessment (MNA) score < 17/30 and/or serum albumin level < 35 g/L)
Depression	Diagnosed by semi-structured interview to identify criteria for a major depressive episode from the Diagnostic and Statistical Manual of Mental Disorders (DSM-IV)
Cognitive impairment	Mini-Mental State Examination score (MMSE) ≤ 23/30
No. of severe (grade 3/4) comorbidities	Assessed by the Cumulative Illness Rating Scale for Geriatrics (CIRS-G; 0,1 ≥2)
Functional impairment	Activities of Daily Living score (ADL) ≤5/6
Age	In two classes: ≤80 years; >80 years
Tumor site	Colorectal, upper gastrointestinal tract and liver, breast, prostate, other urologic malignancies, hematologic malignancies, other
Metastatic status	M0, absence of distant metastases; M1, presence of distant metastases; Mx, metastatic status unknown / NA, not applicable
Status at the time of the GA	In/outpatient

Scoring rules are applied to classify patients into one of four profiles: Class 1, relatively healthy; Class 2, malnourished; Class 3, cognitive and mood impaired; Class 4, globally impaired.

Supplementary Table S3. Variables Included in the Frailty Index (N = 52 health deficits included)

Deficit count	Variable	Code
1	Assistance in bathing	0: No ; 0.5: Little ; 1:Yes
2	Assistance in getting dressed	0: No ; 0.5: Little ; 1:Yes
3	Problems carrying out personal grooming	0: No ; 1:Yes
4	Help using telephone	0: No ; 1:Yes
5	Help taking Medications	0: No ; 1:Yes
6	Help with Finances	0: No ; 1:Yes
7	Toileting problems	0: No ; 0.5: Little ; 1:Yes
8	Urinary incontinence	0: No ; 0.5: Occasionally ; 1:Yes
9	Bulk difficulties	0: No ; 1:Yes
10	Diarrhea	0: No ; 1:Yes
11	Problems for cooking	0: No ; 1:Yes
12	Feeding problems	0: No ; 0.5: Little ; 1:Yes
13	Swallowing difficulties	0: No ; 1:Yes
14	Anorexia (loss of appetite)	0: No ; 0.5: Mild ; 1: Severe
15	Malnutrition	0: No ; 1:Yes
16	Mid-Arm Circumference (cm)	0: >22 ; 0.5: 21-22 ; 1:<21
17	Low Calf Circumference (<31cm)	0: No ; 1:Yes
18	Poor Dentition	0: No ; 1:Yes
19	Recent weight loss	0: No ; 0.5: 1–3kg ; 1: >3kg
20	Assistance getting in and out of a chair or bed	0: No ; 0.5: Little ; 1:Yes
21	Problems walking around home	0: No ; 1:Yes
22	Help with transportation	0: No ; 1:Yes
23	Impaired mobility	0: No ; 1:Yes
24	Musculoskeletal problems	0: No ; 1:Yes
25	Single-leg stance ≥5 seconds	0: No ; 1:Yes
26	Timed Up-and-Go test >20 sec.	0: No ; 1:Yes
27	Fall risk (TUG score)	0: No ; 1:Yes
28	Fall(s) in the 6 past months	0: No ; 1:Yes
29	Mood problems (mini-GDS ≥1)	0: No ; 1:Yes
30	Feeling sad, blue, depressed	0: No ; 1:Yes
31	Asthenia	0: No ; 1:Yes
32	Depression (clinical diagnosis)	0: No ; 1:Yes
33	Memory complains	0: No ; 1:Yes
34	Cognitive disorders	0: No ; 1:Yes
35	Cognitive impairment (MMSE ≤ 23)	0: No ; 1:Yes
36	History relevant to cognitive impairment or loss	0: No ; 1:Yes
37	Neurological problems	0: No ; 1:Yes
38	Hearing difficulty	0: No ; 1:Yes
39	Vision problems	0: No ; 1:Yes
40	Metastases	0: No ; 1:Yes
41	Self-rated health status	0: Good ; 0.5: Fair ; 1: Poor
42	Pain related to cancer or other	0: No ; 1:Yes
43	Sleeping disorders	0: No ; 1:Yes
44	Diabetes mellitus	0: No ; 1:Yes
45	Arterial hypertension	0: No ; 1:Yes
46	Heart failure	0: No ; 1:Yes
47	Coronary Heart Disease	0: No ; 1:Yes

48	Heart rhythm di**s**order	0: No ; 1:Ye**s**
49	Dy**s**lipidemia	0: No ; 1:Ye**s**
50	Chronic re**s**piratory failure	0: No ; 1:Ye**s**
51	Chronic renal failure	0: No ; 1:Ye**s**
52	Chronic liver failure	0: No ; 1:Ye**s**

Supplementary Table S4. G8 screening questionnaire

**Items**	**Score**
Has food intake declined over the past 3 months due to loss of appetite, digestive problems, or chewing or swallowing difficulties?	0: severe decrease in food intake<br>1: moderate decrease in food intake<br>2: no decrease in food intake
Weight loss during the last 3 months	0: weight loss > 3kg<br>1: does not know<br>2: weight loss between 1 and 3 kg<br>3: no weight loss
Mobility	0: bed or chair bound<br>1: able to get out of bed/chair but does not go out<br>2: goes out
Neuropsychological problems	0: severe dementia or depression<br>1: mild dementia or depression<br>2: no psychological problems
Body mass index (BMI weight in kg/height in m^2	0: BMI < 18.5<br>1: BMI = 18.5 to BMI < 21<br>2: BMI = 21 to BMI < 23<br>3: BMI = 23 and >23
Takes more than 3 prescription drugs per day	0: yes<br>1: no
Compared with other people of the same age, how does the patient rate his or her health status?	0: not as good<br>0.5: does not know<br>1: as good<br>2: better
Age	0: > 85 years<br>1: 80-85 years<br>2: < 85 years
**Total Score**	**0–17**

Supplementary Table S5. Modified-G8 screening questionnaire

Items	Score
Weight loss during the last 3 months	10: weight loss > 3kg / does not know<br>2: weight loss between 1 and 3 kg<br>0: no weight loss
Neuropsychological problems	3: mild / severe dementia or depression<br>0: no psychological problems
Takes at least 6 prescription drugs per day	2: yes<br>0: no
Compared with other people of the same age, how does the patient rate his or her health status?	3: not as good / does not know<br>0: as good or better
Performance Status (PS)	12: PS 2, 3 or 4 (ambulatory but unable to carry out any work activities / confined to bed >50% / disabled)<br>4: PS 1 (restricted in physically strenuous activity but ambulatory and able to carry out work of a light or sedentary nature)<br>0: PS 0 (fully active)
Past history of heart failure or coronary artery disease	5: yes<br>0: no
**Total Score**	**0–35**
